# PREVENTING SOCIAL ISOLATION AND LONELINESS AMONG OLDER ADULTS WITH DEMENTIA: FINDINGS AND INSIGHTS FROM JAPAN

**DOI:** 10.1093/geroni/igae098.2482

**Published:** 2024-12-31

**Authors:** Limei Chen, Megumi Inoue

**Affiliations:** George Mason University, Fairfax, Virginia, United States; George Mason University, Fairfax, Virginia, United States

## Abstract

This study examines the implementation and impact of social capital interventions to mitigate the interplay between social isolation, loneliness, and dementia within Japan’s aging population. With an emphasis on advancing knowledge through a socio-ecological approach, our research focuses on social capital interventions, leveraging “chiiki-ryoku” (community power) at the community level for individuals with dementia and their caregivers. These interventions include Community (Dementia) Cafes, Dementia Supporter Caravan Training Programs, Community Social Workers, and Dementia Friendly-Communities, which are evaluated for their effectiveness in fostering social engagement and support networks. Our findings highlight the critical role of social capital in enhancing community resilience and well-being, demonstrating that interventions that cultivate active social participation and strong social networks in defined physical spaces such as junior high school area or municipal area can serve as protective factors against social isolation and loneliness. The research contributes to the broader discourse on social capital and dementia care by revealing the nuanced challenges and potential for meaningful impact through context-sensitive, integrative strategies. It posits that fostering a sense of belonging and meaningful relationships within communities can significantly alleviate the adverse effects of loneliness and social isolation among those affected by dementia. Through a detailed examination of Japan’s response to dementia-related social isolation, this study provides insights into the development of effective, sustainable interventions that promote social health and well-being in aging societies.

